# Epidemiologic Findings from Case Investigations and Contact Tracing for First 200 Cases of Coronavirus Disease, Santa Clara County, California, USA

**DOI:** 10.3201/eid2705.204876

**Published:** 2021-05

**Authors:** Nancy Ortiz, Elsa Villarino, James T. Lee, Kristina L. Bajema, Jessica N. Ricaldi, Shanon Smith, Wen Lin, Margaret Cortese, Albert E. Barskey, Juliana F. Da Silva, Brandon J. Bonin, Sarah Rudman, George S. Han, Marc Fischer, Shua J. Chai, Sara H. Cody, for the Santa Clara County COVID-19 Case Investigation Team

**Affiliations:** Centers for Disease Control and Prevention, Atlanta, Georgia, USA (N. Ortiz, J.T. Lee, K.L. Bajema, J.N. Ricaldi, M. Cortese, A.E. Barskey, J.F. Da Silva, M. Fischer, S.J. Chai);; County of Santa Clara Public Health Department, San Jose, California, USA (E. Villarino, S. Smith, W. Lin, B.J. Bonin, S. Rudman, G.S. Han, S.H. Cody)

**Keywords:** COVID-19, coronavirus disease, SARS-CoV-2, severe acute respiratory syndrome coronavirus 2, viruses, respiratory infections, zoonoses, epidemiology, California

## Abstract

In January 2020, Santa Clara County, California, USA, began identifying laboratory-confirmed coronavirus disease among residents. County staff conducted case and contact investigations focused on households and collected detailed case demographic, occupation, exposure, and outcome information. We describe the first 200 test-positive cases during January 31–March 20, 2020, to inform future case and contact investigations. Probable infection sources included community transmission (104 cases), known close contact with a confirmed case-patient (66 cases), and travel (30 cases). Disease patterns across race and ethnicity, occupational, and household factors suggested multiple infection risk factors. Disproportionately high percentages of case-patients from racial and ethnic subgroups worked outside the home (Hispanic [86%] and Filipino [100%]); household transmission was more common among persons from Vietnam (53%). Even with the few initial cases, detailed case and contact investigations of household contacts capturing occupational and disaggregated race and ethnicity data helped identify at-risk groups and focused solutions for disease control.

On January 31, 2020, the Santa Clara County Department of Public Health (SCCDPH) in San Jose, California, USA, identified its first case of coronavirus disease (COVID-19) in a resident who had recently returned from Wuhan, China (*1*). On February 28, the county reported its first case of COVID-19 associated with probable community transmission, 48 hours after the first presumed community-acquired case in the United States was identified 91 miles north in Solano County (*2*). Staff of the SCCDPH, the California Department of Public Health, and the Centers for Disease Control and Prevention (CDC) began conducting detailed interviews with each case-patient or their surrogate to identify, quarantine, and monitor close contacts, and isolate and test those who were symptomatic. Santa Clara initiated a series of community mitigation strategies to slow the spread of the virus that causes COVID-19, severe acute respiratory syndrome coronavirus 2 (SARS-CoV-2), including canceling large gatherings (*3*,*4*). On March 16, Santa Clara and 5 adjacent San Francisco Bay Area counties became the first US region to implement shelter-in-place orders requiring all residents to limit activity outside of their home and to order nonessential businesses and operations to close (*5*). SCCPHD collected detailed information on demographic characteristics to help identify communities at risk and those disproportionately affected by COVID-19. Since the initial identification of cases, surges in COVID-19 incidence have often constrained public health and community capacity to respond, including overwhelming case and contact investigation efforts. We describe the epidemiology of the first 200 COVID-19 cases reported to SCCPHD to identify key transmission factors that could already be identified early in the COVID-19 pandemic through detailed case investigation and contact tracing focused on households and to demonstrate the utility of focusing these efforts throughout the pandemic response.

## Methods

### Case Identification and Testing

We defined a confirmed COVID-19 case as an illness in a resident of Santa Clara County with SARS-CoV-2 detected by reverse transcription PCR (RT-PCR) on a nasopharyngeal or oropharyngeal swab specimen by a public health, hospital, or reference clinical microbiology laboratory or CDC. Testing was recommended in line with the following evolving CDC Person Under Investigation case definition: clinical findings of lower respiratory illness and travel to a Wuhan, China (later expanded to all of China) or an epidemiologic link to a laboratory-confirmed COVID-19 case (*6*,*7*); hospitalization for severe respiratory disease and no alternative diagnosis (*8*); and clinically compatible illness regardless of travel or known contact with a confirmed case-patient. Included COVID-19 case-patients comprised those reported to SCCDPH and those identified by a community-based sentinel surveillance project for COVID-19 conducted during March 5–14, 2020, among clinic patients with respiratory illness who tested negative for influenza virus (*9*). This activity was reviewed by CDC and was conducted consistent with applicable federal law and CDC policy.

### Case Investigation and Contact Tracing

SCCDPH, California Department of Public Health, and CDC staff identified cases reported to California’s electronic reportable disease system. Staff interviewed COVID-19 cases or their surrogates for information on case age, sex, race, ethnicity, address, occupation, travel history, known contact with another confirmed case-patient, symptom onset (earliest of any symptoms listed on CDC’s standardized case report form) (*10*), and hospitalization. Investigators collected detailed race and ethnicity data, including racial subgroup among case-patients reporting Asian ancestry.

Case-patients with no recent travel and no known close contact with another confirmed case-patient in the 2 weeks before symptom onset were classified as probable community transmission. Known close contact was defined as living with, caring for, working with, transporting, or prolonged exposure (close contact <6 feet for >30 minutes) to a person with confirmed COVID-19. Case-patients with any travel outside of Santa Clara County in the 2 weeks before their symptom onset were considered travel-associated cases.

SCCPHD’s contact tracing involved identifying persons with close contact with the case-patient 2 weeks after the case-patient’s symptom onset and notifying contacts of their exposure. Owing to the rapid rise in case counts and limited personnel capacity, the team focused on following up with household contacts. In-hospital outcomes were collected from review of medical records and case-patient interviews. Deaths through May 20, 2020 (60 days after the 200th case was reported), were defined as COVID-19–associated if the cause or other contributing cause on the death certificate was listed as COVID-19.

### Data Analysis

We collected data using standard forms and open-ended case-patient interviews and entered results into Excel 365 (Microsoft, https://www.microsoft.com) and California’s electronic reportable disease system. Categorical variables were described as counts and percentages, and continuous variables were described using median and range. We estimated associations between illness severity measures (hospitalization defined as admission for >1 night in an inpatient acute-care facility [including intensive-care unit (ICU) stay and mechanical intubation with ventilation]; ICU stay [including mechanical ventilation]; mechanical ventilation; and death) as the dependent variables, and age and sex as independent variables with odds ratios (ORs) and 95% CIs using bivariate logistic regression. Because of the limited number of cases, to avoid invalid results or unstable models, measurements were not adjusted. We analyzed data using Stata 14 (StataCorp, https://www.stata.com) and Epi Info version 7 (Epi Info, https://www.cdc.gov/epiinfo) and generated maps using Excel 365 (Microsoft, https://www.microsoft.com).

## Results

### Case Description

**Figure 1:**
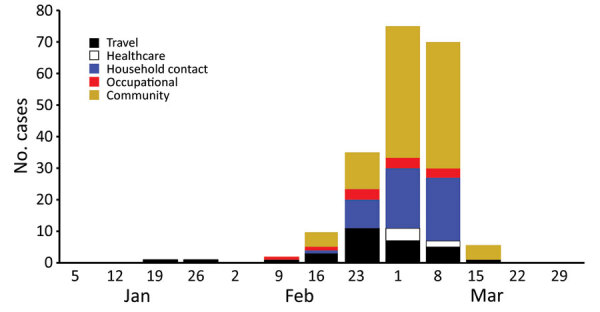
Week of symptom onset, for first 200 confirmed coronavirus disease cases, by exposure source, Santa Clara County, California, USA, January 31–March 20, 2020.

Of the 200 cases with laboratory confirmation of SAR-CoV-2 positivity during January 31–March 20, 2020, a total of 191 (96%) were identified through routine surveillance and contact tracing and 9 (4%) were identified through clinic-based sentinel surveillance. Onset of illness ranged from January 24 through March 18; these case-patients were exposed before shelter-in-place orders were invoked (Figure 1). Among the first cases identified during January 31–February 2, travel accounted for the largest reported source of exposure. Over subsequent weeks, case-patients reported unknown and household exposure at higher frequencies than other exposures. The percentage of case-patients who were hospitalized decreased over time as testing availability increased and focus of testing broadened to include additional populations, including symptomatic contacts.

**Figure 2:**
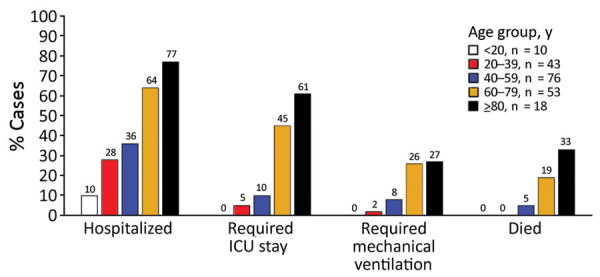
Hospitalization status and outcomes, for first 200 confirmed coronavirus disease cases, by age group, Santa Clara County, California, USA, January 31–March 20, 2020. Outcomes are classified by most severe status at time of case investigation. Deaths are as of May 20, 2020. ICU, intensive-care unit.

Among the 200 case-patients, 112 (56%) were male, and the median age was 50 years (range 6 months–94 years); only 10 (5%) case-patients were <20 years of age, whereas 71 (36%) were >60 years of age (Table). The racial and ethnic distribution of case-patients was similar to that of the county population overall: 70 (35%) reported as Asian, 52 (26%) Hispanic, 52 (26%) White non-Hispanic, 4 (2%) Black non-Hispanic, and 3 (1%) Pacific Islander; race or ethnicity was unknown for 19 (9%) case-patients. Although Asian-identifying persons comprised a similar proportion of case-patients as that of Santa Clara County, a higher proportion of case-patients identified as Filipino (10% vs. 5%), a similar proportion as Vietnamese (7% vs. 7%), and a lower proportion as Indian (4% vs. 9%) or Chinese (4% vs. 10%) than among the general population of Santa Clara County (*11*,*12*). Of the 200 case-patients, 89 (44%) were hospitalized (Table); 45 (23%) were on a general ward, 18 (9%) were admitted to an ICU without requiring mechanical ventilation, and 26 (13%) required mechanical ventilation in an ICU. The proportion of case-patients hospitalized, admitted to the ICU, requiring mechanical ventilation, and who died each increased with increasing age (Figure 2). Compared with case-patients <60 years of age, case-patients >60 years of age had higher odds of hospitalization (OR 4.4 [95% CI 2.4–8.3]), ICU stay (OR 10.9 [95% CI 4.9–24.2]), mechanical ventilation (OR 6.3 [95% CI 2.5–16.0]), and death (OR 9.0 [95% CI 2.9–28.4]). No statistically significant association was observed between clinical outcomes and sex.

**Table:** Characteristics of first 200 confirmed COVID-19 case-patients, Santa Clara County, California, USA, January 31–March 20, 2020*

Characteristic	No. (%) COVID-19 cases
Sex	
M	112 (56.0)
F	88 (44.0)
Age group, y	
<20	10 (5.0)
20–39	43 (21.5)
40–59	76 (38.0)
60–79	53 (26.5)
>80	18 (9.0)
Race/ethnicity	
Asian	70 (35.0)
Filipino	20 (10.0)
Vietnamese	15 (7.5)
Chinese or Taiwanese	9 (4.5)
Indian	8 (4.0)
Korean	1 (0.5)
Laotian	1 (0.5)
Unknown	16 (8.0)
Hispanic	52 (26.0)
White non-Hispanic	52 (26.0)
Pacific Islander	3 (1.5)
Black or African American	4 (2.0)
Unknown	19 (9.5)
Exposure type	
Probable community transmission	104 (52.0)
Close contact with known case-patient	
Household	49 (24.5)
Occupational	17 (8.5)
Travel	
International	19 (9.5)
Middle East	5 (2.5)
Asia	4 (2.0)
Europe	3 (1.5)
Central America	1 (0.5)
Multinational cruise	6 (3.0)
Within the United States	11 (5.5)
Occupation	
Perform work outside the home	82 (41.0)
Healthcare worker	15 (7.5)
Food service Worker	8 (4.0)
Construction worker	7 (3.5)
Airport worker	6 (3.0)
Grocery store worker	5 (2.5)
Firefighter	5 (2.5)
Daycare worker	4 (2.0)
Janitor	3 (1.5)
Housekeeper	3 (1.5)
Retail worker	3 (1.5)
Other (e.g., security guard or realtor)†	23 (11.5)
Retired	34 (17.0)
Perform work inside the home	25 (12.5)
Software technology or engineer	9 (4.5)
Other (e.g., business, finance, or executive)†	16 (8.0)
Unemployed	13 (6.5)
Child (<18 y)	7 (3.5)
Student	5 (2.5)
Unknown	34 (17.0)
Hospitalization status‡ and outcome	
Not hospitalized	111 (55.5)
Hospitalized	89 (44.5)
Did not require ICU stay	45 (22.5)
Required ICU stay, but did not require mechanical ventilation	18 (9.0)
Required ICU stay and required mechanical ventilation	26 (13.0)
Died	20 (10.0)

*COVID-19, coronavirus disease; ICU, intensive care unit. †Occupations reported by <2 case-patients. ‡Classified by most severe status at time of case investigation.  ¶As of March 20, 2020. Deaths for which COVID-19 was listed as the cause or other contributing cause on the death certificate were defined as COVID-19-associated deaths.

Among the 200 case-patients, 20 (10%) had a matching death certificate. The median age of deceased case-patients was 70.5 years (range 42–87 years), and 15 (75%) were male. Among the 20 case-patients who died, 9 (45%) were Asian, 5 (25%) were White non-Hispanic, 2 (10%) were Hispanic, and 4 (20%) had unknown race or ethnicity. Five (25%) of the 20 deaths occurred among persons of Filipino ethnicity; these case-patients did not have a known close contact to one another.

**Figure 3:**
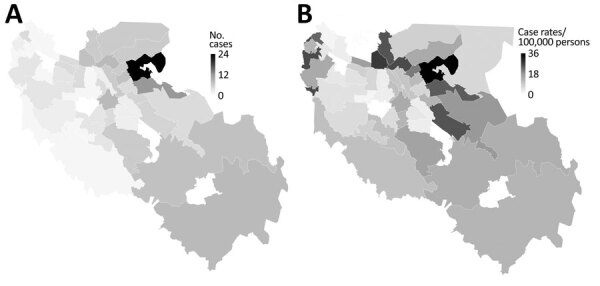
Geographic location of first 200 confirmed coronavirus disease cases, by case-patient’s ZIP code area of residence (for those areas with >2,000 residents), Santa Clara County, California, USA, January 31–March 20, 2020. A) No. cases; B) case rate (cases/100,000 population).

Case-patient residences were distributed among 47 (79%) of the 59 ZIP codes in the county; 18 (30%) ZIP codes had 1–2 cases, 13 (22%) had 3–4 cases, and 16 (8%) had >5 cases (Figure 3, panel A). Case-patient residences clustered in the northeastern part of the county, where 2 adjacent ZIP codes accounted for 36 (18%) of the 200 case-patients; in the ZIP code with the most cases, 9 were associated with a single household. COVID-19 incidence rates by ZIP code ranged from 0–113 cases/100,000 persons; rates were generally highest in eastern ZIP codes in the county (Figure 3, panel B).

### Exposure Type and Setting

Of the 200 case-patients, 66 (33%) had known close contact with another confirmed case-patient, 30 (15%) were considered travel-associated cases (Table), and 104 (52%) were attributable to probable community transmission. Among the 66 case-patients with known close contact with another confirmed case-patient, 49 (74%) were exposed to a household member, and 17 (26%) had occupational exposures. Most households with evidence of transmission (13/15 [86%]) had 2–3 confirmed case-patients identified. However, 2 multigenerational households each had 9 and 4 case-patients; the cluster of 4 case-patients was only identified as a result of contact tracing.

Of the 200 case-patients, 159 (79%) were adults with reported occupation. Of these, 111 (69%) were actively employed (not retired and reported employment), and of these, 82 (73%) reported jobs requiring work outside the home, which included healthcare workers, firefighters, food service workers, retail employees, construction workers, housekeepers, and other workers. Among these 82 case-patients reporting jobs requiring work outside the home, 46% of exposures were attributable to probable community transmission, followed by 22% household and 21% occupational exposures.

Type and location of exposure, as well as having an occupation that requires work outside the home, varied by race and ethnicity. Among 49 cases in Hispanic adults, occupation was known for 44 (89%); of the 38 actively employed, 89% held occupations that required them to work outside of the home. Occupation was known for 16 of 20 Filipino case-patients; for the 9 case-patients who were actively employed, all had jobs outside the home. Occupational exposure to a confirmed case-patient, including in a healthcare setting, accounted for 5 (25%) of 20 cases in Filipino persons, compared with 12 (7%) of all other cases with reported race and ethnicity. Household transmission accounted for exposures in 53% of Vietnamese case-patients and 32% of Hispanic case-patients, compared with 23% of all other case-patients with known race/ethnicity. Among case-patients of Indian and Chinese ethnicity, >50% had travel-related exposures.

Among the 17 case-patients with an occupational exposure to a confirmed case-patient, 11 (64%) exposures occurred in a nonhealthcare setting. Of these 11, all were employed as essential workers in occupations or settings in which they had frequent contact with many persons in the community. Occupational clusters and groupings included 6 airport employees, 4 employees at a supermarket, 3 childcare workers who shared a classroom and bathroom, and 2 firefighters who worked at the same station. At least 3 additional cases were identified among other firefighters who worked at the same station or attended a common function but were not Santa Clara County residents.

Of the 200 case-patients, 16 (8%) were healthcare workers with jobs that provided direct patient care or were first responders with direct patient exposure, of whom 8 (50%) were nurses. Only 6 transported, cared for, or had other known close contact with a confirmed case-patient in a healthcare setting. Of the other 10 cases in healthcare workers, 1 case-patient had travel-related exposure, 3 had known close contact with a case-patient in their household, and 6 did not have exposure to a known COVID-19 case-patient and were categorized as attributable to probable community transmission.

## Discussion

Detailed case investigations and household contact tracing of the first 200 case-patients of COVID-19 in Santa Clara County were able to help elucidate factors associated with being a COVID-19 case-patient and identify populations at risk for infection early in the response, including possible racial and ethnic disparities, elevated risks within households, and high-risk occupational groups. Many of these factors and populations at risk were subsequently confirmed by studies later in the pandemic (*13*,*14*). Case investigations identified possible sources of transmission in 96 (48%) of cases, and for those case-patients with known exposure, household transmission was the most commonly reported source, especially in Vietnamese and Hispanic communities. Work outside the home was commonly reported by Hispanic case-patients. Case-patients >60 years of age had significantly higher odds of being hospitalized, being admitted to the ICU, requiring mechanical ventilation, and dying; these findings are consistent with reports from China, Italy, and other parts of the United States (*15*–*17*).

Because SCCPHD conducted contact tracing and monitoring specifically among household contacts of case-patients, the finding that approximately one quarter of the first 200 case-patients were household contacts of a confirmed case-patient is not surprising. However, SCCPHD’s prioritization of contact tracing and monitoring contacts within households early in the pandemic was high-yield, and findings were consistent with disease transmission factors for COVID-19 reported in subsequent studies (*18*,*19*). Investigations identified not only that older persons had increased odds of poor outcomes from COVID-19 but also that case-patients with multiple factors potentially increased risk. For example, several large clusters were identified within families that consisted of members of multiple generations, and several individuals >80 years of age might have been exposed. In 2 of these clusters, the index case-patient was a nonelderly household member who presumably transmitted SARS-CoV-2 to elderly household members. Anecdotally, several of these households also reported crowding and inability to self-isolate from other members within the home (Santa Clara COVID-19 Case Investigation Team, pers. comm., group discussion during case review, March 2020). Households have been identified as a high-risk setting for SARS-CoV-2 transmission (*20*–*22*), and household crowding is a risk factor for COVID-19 (*23*). In the ZIP code with the highest case rate in northeast Santa Clara, 14% of households are overcrowded (>1.0 persons/room), as measured by the American Community Survey, compared with the median of 6% of households in Santa Clara County as a whole (*24*). Although information on an individual case-patient’s household density was not collected as part of case and contact investigations, 4 (33%) of 12 ZIP codes where household transmission was identified reported >10% frequency of overcrowded households, compared with 7 (20%) of 35 ZIP codes where cases were identified but no household transmission was noted. Household density might be associated with other factors, such as high-risk occupations of household members (*25*,*26*), to increased risk for COVID-19 within households. Case investigators collecting information regarding household density during interviews can help not only to elucidate transmission risk in a particular household, but also link persons at high risk for poor outcomes to resources to prevent household transmission. One example of a solution to prevent household transmission is The NYC Test and Trace Corps, a collaborative public health program led by NYC Health + Hospitals in collaboration with the New York City Department of Public Health and Mental Hygiene, which offers hotel stays for persons who have COVID-19, exhibit COVID-19 symptoms, or are contacts of a known COVID-19 case-patient and who need to isolate or quarantine from household members (*27*).

Working outside the home, especially with public-facing duties (e.g., airport workers), was especially common in this early cohort; >40% of case-patients reported an occupation that did not allow them to work from home. A large frequency of case-patients who performed work outside the home did not report a known exposure or travel, suggesting that difficult-to-trace exposures, such as exposure to someone the case-patient did not know or did not know was infected, probably occurred (*28*). Moreover, occupational exposures were probably more common than we reported, because case-patients who did not have known exposure to a person with confirmed COVID-19 and had not traveled were classified as having community exposure. Identifying the source of exposure for case-patients with occupations that interact with the public might prove to be very labor-intensive or impossible, given the number of potential contacts involved. However, case and contact investigations, at a minimum, should include notifying co-workers and alerting employers to a positive case in a workplace (*29*) and collecting occupation data to help identify occupational subgroups at risk.

Occupational exposures probably differed by racial and ethnic groups among the first 200 case-patients in Santa Clara County. Among employed Filipino case-patients, all held jobs that required work outside the home. Although few Hispanic case-patients reported an occupational exposure with a confirmed COVID-19 case-patient, a greater percentage of Hispanic case-patients (89%) had occupations that required them to work outside the home than did White non-Hispanic case-patients (56%). Many of the Hispanic case-patients in Santa Clara County communicated that they could not afford the lost wages that would result from staying home from work (Santa Clara COVID-19 Case Investigation Team, pers. comm., group discussion during case review, March 2020). Hispanic persons nationwide have reported higher frequencies of job loss and wage reduction because of the COVID-19 pandemic compared with persons from other racial and ethnic minority groups, and less than one third of Hispanic persons surveyed reported that they could weather a financial emergency (*30*). These financial and occupational factors together might be critical drivers for transmission within the Hispanic population in Santa Clara County and perhaps statewide, where Hispanic persons have accounted for a disproportionately high number of cases (*31*). A disproportionately high percentage of COVID-19 cases and deaths occurred in Filipino persons; cases among Filipino persons associated with occupational exposures involved providing direct patient care to known COVID-19 patients or contact with a person with confirmed COVID-19 in public-facing service jobs.

Household exposures also differed by racial and ethnic groups. Vietnamese and Hispanic case-patients more frequently reported exposure to a person with confirmed COVID-19 in their household compared with case-patients from other race and ethnicity groups. Anecdotally, among Vietnamese and Hispanic case-patients, >3 reported living in multigenerational households with high densities of persons and an inability to self-isolate within the home, posing a serious risk to older adults residing in these households. Household case clusters occurred in eastern ZIP codes that had high percentages of Hispanic persons (58% of the population in the ZIP code with the most cases and highest rates) and Vietnamese persons (22%), compared with 26% of Hispanic and 7% of Vietnamese persons in the county as a whole (*32*–*37*). Together, these findings suggest that household crowding might be an especially important driver of household transmission in traditionally underserved communities.

Few of the first 200 COVID-19 cases in Santa Clara County occurred in healthcare workers or persons in institutional or congregate living settings. Although more than one third of infected healthcare workers reported an occupational exposure and a quarter traveled or had a nonoccupational close-contact exposure, none of these exposures was identified for 40% of them. Evidence to date does not support substantial occupational transmission of SARS-CoV-2 to healthcare workers (*38*). Community transmission could have been an important source of exposure for healthcare workers, given the widespread community transmission occurring simultaneously in Santa Clara County.

One limitation of this analysis is, as with most reports on COVID-19, case identification was largely dictated by testing practices. At the start of the outbreak, the number of persons eligible for testing according to CDC criteria and testing capacity were limited, biasing these initial findings to case-patients with higher disease and mortality rates and to persons with recent travel or known contact with a confirmed case-patient. Had testing been more widely available and criteria included milder symptoms or risk for exposure regardless of symptoms, broader or earlier detection of community transmission might have occurred. This investigation occurred when information was limited for this new and emerging disease. The definition of prolonged COVID-19 exposure and guidance for case and contact investigations has been updated since this investigation concluded (*39*). Although we observed differences in sources of exposure by race and ethnicity, data on race were missing for 19 (9%) cases and racial subgroup for 16 (23%) of 70 cases among Asian persons; therefore, these data should be interpreted with caution. Our data reflect the epidemiology of COVID-19 in Santa Clara early in the pandemic among those with clinical manifestations that were eligible for testing and probably are not reflective of the current epidemiology (*40*).

Even with results from only the first 200 case-patients, detailed case investigation and contact tracing focused on households revealed patterns of at-risk populations, including older age adults, racial and ethnic subgroups, occupational categories, and potentially crowded households. Detailed case reviews, including disaggregation of race and ethnicity data, helped identify local factors of transmission and disparities important for public health intervention. Importantly, occupational exposures continue to be a source of infection (*41*), and understanding transmission risk within specific occupational settings, especially among professions that require persons to work outside their homes, is important to ensure safe workplaces and reopening of economies as the pandemic continues to evolve. As mitigation measures to suppress community transmission evolve throughout the pandemic response, novel preventive measures (e.g., temporary housing) might continue to be necessary to protect disproportionately affected subpopulations and older adults.
